# Cell Penetrating Peptides in the Delivery of Biopharmaceuticals

**DOI:** 10.3390/biom2020187

**Published:** 2012-03-30

**Authors:** Were LL Munyendo, Huixia Lv, Habiba Benza-Ingoula, Lilechi D. Baraza, Jianping Zhou

**Affiliations:** 1 Department of Pharmaceutics, China Pharmaceutical University, 24 Tongjiaxiang, Nanjing 210009, Jiangsu, China; Email: munyendollw@yahoo.com (W.L.L.M); lvhuixia@163.com (H.L.); iamzhoujianping@163.com (Z.J.); 2 Institut de Chimie Pharmaceutique Albert Lespagnol (Lille 2) (ICPAL), 3 rue du Professeur Laguesse 59006, France; Email: biba0707@live.com; 3 Department of Pure and Applied Chemistry, Masinde Muliro University of Science and Technology, P.O. Box 190-50100, Kakamega, Kenya; Email: danstone_baraza@yahoo.com

**Keywords:** cell penetrating peptides, oligoarginines, carriers, drug delivery

## Abstract

The cell membrane is a highly selective barrier. This limits the cellular uptake of molecules including DNA, oligonucleotides, peptides and proteins used as therapeutic agents. Different approaches have been employed to increase the membrane permeability and intracellular delivery of these therapeutic molecules. One such approach is the use of Cell Penetrating Peptides (CPPs). CPPs represent a new and innovative concept, which bypasses the problem of bioavailability of drugs. The success of CPPs lies in their ability to unlock intracellular and even intranuclear targets for the delivery of agents ranging from peptides to antibodies and drug-loaded nanoparticles. This review highlights the development of cell penetrating peptides for cell-specific delivery strategies involving biomolecules that can be triggered spatially and temporally within a cell transport pathway by change in physiological conditions. The review also discusses conjugations of therapeutic agents to CPPs for enhanced intracellular delivery and bioavailability that are at the clinical stage of development.

## 1. Introduction

Cell penetrating peptides (CPPs) are short peptide sequences that are able to transport molecules across the cell membrane. The CPPs, also known as protein transduction domains (PTDs) are made up of three to 30 peptide residues [1]. They are employed to enhance extracellular and intracellular internalization of various biomolecules including plasmid DNA, siRNA, oligonucleotide, peptide-nucleic acid (PNA), peptides, proteins and liposomes [2].

The notion of protein transduction domains (PTD) was proposed based on the observation that some proteins, mainly transcription factors, could shuttle within the cell and from one cell to another [3]. The first observation was made in 1988 when it was shown that the transcription transactivating (Tat) protein of HIV-1 could enter cells and translocate into the nucleus. In 1991, *Drosophila antennapedia* homeodomain was illustrated to be internalized by neuronal cells. This was the origin of the discovery in 1994 of the first PTD or CPP, a 16-merpeptide derived from the third helix of the homeodomain of Antennapedia termed Penetratin (RQIKIYFQNRRMKWKK) [4]. In 1997, the first non-covalent CPP for delivery of nucleic acids MPG was designed which was closely followed by development of Pep-1 for non-covalent cellular delivery of proteins and peptides. This was followed by the identification of the minimal peptide sequence of TAT (47YGRKKRRQRRR57) required for cellular uptake in 1998 [4].

Proofs-of-concept of the CPPs *in vivo* application for the delivery of small peptides and large proteins and for delivery of PNAs using the chimeric peptide Transportan, derived form the N-terminal fragment of the neuropeptide galanin, linked to mastoparan, a wasp venom peptide was a great breakthrough. Other CPPs that could trigger the movement of a therapeutic agent across the cell membrane into the cytoplasm are continually being designed [5,6].

## 2. Classification of Cell Penetrating Peptides

There is no unified classification of CPPs, however they could be divided into two broad classes.

### 2.1. Classification based on Linkage with Therapeutic Agent

Based on the interaction between cell penetrating peptides and the therapeutic agent two main sub-classes of CPPs can be distinguished. The first sub-class includes CPPs that are chemically linked to the therapeutic agents while the second sub-class encompasses those that form stable non-covalent complexes with the therapeutic agents [4].

Apart from these two main sub classes in the recent past, another method for non-covalent coupling of CPPs for a nucleic acid cargo (*i.e.*, antisense oligomer, siRNA) has been evidenced. This entails sequence complementary hybridization, for instance the binding of the oligonucleotide NFkappaB decoy to the nonamer PNA sequence that resulted in a stable complex that was efficiently translocated across the plasma membrane [7].

#### 2.1.1. Covalent Bonded CPPs

This sub-class of CPPs forms a covalent conjugate with the therapeutic agent by chemical cross-linking or by cloning followed by expression of a CPP fusion protein [8]. Such interactions have been realized in several CPPs including peptides derived from Tat, penetratin, polyarginine peptide Arg_8_ sequence, Transportan, VP22 protein from Herpes Simplex Virus (HSV), antimicrobial peptides Buforin I and SynB as well as polyproline sweet arrow peptide [9,10,11]. This is further ascertained from the report of Schwarze and group confirming that several proteins do traverse biological membranes through protein transduction. The small sections of these proteins (10–16 residues long) were noted to be responsible for linking these domains covalently to the compounds, peptides, antisense peptide nucleic acids or 40-nm iron beads, or as in-frame fusions with full-length proteins [12].

A variant of this sub-class has been reported in the recent past; new generations of CPPs which combine different transduction motifs or transduction domains in tandem with protein or oligonucleotide-binding domains [13]. Different explanations have been proposed for this stable but cleavable conjugates that involve mainly disulfide or thio-esters linkages [14]. 

#### 2.1.2. Non-covalent Bonded CPPs

These are CPPs that form non-covalent complexes with biomolecules thereby improving their delivery into cells. They occur mostly as short amphipathic peptide carriers consisting of a hydrophilic (polar) domain and a hydrophobic (non-polar) domain. The amphipathic character arises from either the primary structure or the secondary structure [5]. Primary amphipathic peptides have a sequential assembly of hydrophobic and hydrophilic residues. Secondary amphipathic peptides are formed by the conformational state that allows the positioning of hydrophobic and hydrophilic residues on opposite sides of the molecule [15]. Pep-1 and MPG are primary amphipathic peptides, which form stable complexes with oligonucleotide or protein/peptide through non-covalent electrostatic and hydrophobic interactions [16].

Several studies have reported this kind of non-covalent interaction between CPPs and the therapeutic agent. Amphipathic peptide, Pep-1, was used for the delivery of small peptides and proteins while MPG has been shown to efficiently deliver siRNA into cultured cell lines [17].

### 2.2. Classification based on Chemical Charge Induced by CPP

This classification of CPPs is based on their ionic properties. In regard to the ionic charge induced by the CPP, this class could be subdivided into cationic or amphipathic CPPs.

#### 2.2.1. Cationic

The cationic CPPs essentially contain clusters of polyarginine in their primary sequence. TAT, the basic domain of HIV-1 TAT protein is a typical example of the cationic CPPs. It contains clusters of arginine and lysine residues. Other peptides derived from protein transduction domains include the Penetratin, and VP22 HSV-1 structural protein [18,19].

#### 2.2.2. Amphipathic

These are the MAP and Transportan groups of cell penetrating peptides. The amphipathic class comprises peptides with a high degree of amphipathicity. The high degree of amphipathicity is usually contributed by lysine residues when present as a building block. Other examples include the MPG, Pep-1, MAP，SAP Proline-rich motifs and PPTG1 as a result of hydrophiliity / hydrophobicity of the CPP’s building blocks [20].

## 3. Cell Penetrating Peptides in Biopharmaceuticals

Therapeutic agents are usually delivered intracellularly to exert their therapeutic action inside the cytoplasm or individual organelles such as the nuclei for gene therapy, delivery of deficient lysosomal enzymes in lysosomes for disease therapy, and proapoptotic anticancer drugs in mitochondria for cancer therapy. The cell membrane prevents proteins, peptides, and drug carriers from entering cells unless an active transport mechanism is involved [21]. Cell penetrating peptides therefore are used to promote the delivery of associated drugs and drug carriers into cells. Despite the fact that the exact mechanisms underlying the internalization of CPPs across the cellular membrane are not fully understood, a large number of different therapeutic agents have been efficiently delivered by CPPs. These range from small molecules to proteins and even liposomes and magnetic particles [11]. Typical applications of CPPs in the delivery of biopharmaceutical agents include:

### 3.1. Gene Delivery

This involves delivering therapeutic genes into the nucleus of target cells to achieve expression of a deficient or incorrectly expressed gene product. Difficulties in developing safe and efficient gene delivery vectors that could sustain gene expression for long periods, poor permeability of the plasma membrane of eukaryotic cells to DNA results in low concentration of DNA and other oligonucleotides at their targets [22]. To overcome this, peptide carriers such as polylysine and polyarginine that have membrane-destabilizing properties and could bind with DNA mainly through electrostatic interaction have been developed to facilitate gene transfer into cultured cells and cells in living organisms [23]. For example amphipathic peptides with pH-dependent fusogenic and endosomolytic activities such as the fusion peptide of HA_2_ subunit of influenza hemaglutinin, or synthetic analogs GALA, KALA, JTS1, and histidine-rich peptides have been shown to increase transfection efficiency when associated with poly-L-lysine/DNA, condensing peptide/DNA, cationic lipids, poly-ethyleneimine or polyamidoamine cascade polymers [15].

The ability to condense DNA and to favor endosomal escape by CPPs like PPTG1, and the prevention of endosomal uptake by MPG has led to their use for gene delivery in cultured cells. PPTG1 has been reported as an important component for in *in-vivo* gene expression following intravenous injection [24].

Cationic liposomes, nanoparticles, cationic polymers, and CPPs have also been used as non-viral vectors in preference to the viral vectors. This is because viral vectors, despite their sustained levels of transduction and in some cases efficient and stable integration of exogenous DNA into a wide range of host genomes [25], have been noted to have some disadvantages such as immunogenicity, toxicity, difficulty of large-scale production, hence the preference of non-viral vectors for plasmid DNA delivery [22].

### 3.2. siRNA Delivery

CPPs easily conjugate covalently or non-covalently with siRNAs. The siRNAs covalently linked to Transportan and Penetratin have been associated with a silencing response. Non-covalent complexes or aggregates formed with siRNA usually have a net positive charge when there is a surplus of positive charges over negative charges. The covalent linkage of CPPs to siRNAs results in the formation of small, monomeric CPP/siRNA conjugates of known stoichiometry with high reproducibility [26]. CPPs could therefore be used for delivery of siRNAs either by covalent or non-covalent approaches [27]. However non-covalent strategies are more efficient for siRNA delivery, for example MPG peptide has been extensively reported to improve siRNA delivery into a large panel of cell lines including adherent cell lines, cells in suspension, cancer and challenging primary cell lines biological response [28,29]. It has also been applied for *in vivo* delivery of siRNA targeting OCT-4 into mouse blastocytes. It has equally been used for the delivery of siRNA targeting an essential cell cycle protein, cyclin B1. Intravenous injection of MPG/cyclin B1 siRNA particles has been shown to efficiently block tumor growth [30].

TAT peptide associated with an RNA-binding motif has been reported to block *in vivo* epidermal growth factor (EGF) factor. The CPP complex, cholesterol-Arg_9_ was also shown to enhance siRNA delivery *in vivo* against vascular endothelial growth factors. A small peptide derived from rabies virus glycoprotein linked to polyarginine R_9_ has been reported recently to deliver siRNA in the CNS [31].

A new cell-penetrating peptide, PepFect14 (PF14), which efficiently delivers splice-correcting oligonucleotides (SCOs) to different cell models including HeLa pLuc705 and mdx mouse myotubes; a cell culture model of Duchenne’s muscular dystrophy (DMD) was illustrated in a recent study as a new chemically modified CPP, PF14. Starting from stearyl-TP10, ornithines were utilized as the main source of positive charges instead of lysines. The superior efficiency of poly-L-ornithines was related to the higher affinity for DNA and the ability to make more stable complexes at lower charge ratios [32].

These studies show that CPPs are certainly among the most promising candidates in the development of siRNA-based therapeutics.

### 3.3. Antisense Oligonucleotide Delivery

Antisense technology is based on the use of sequence specific oligonucleotides (ONs) that can hybridize with complementary mRNA strands to cause translational arrest or mRNA degradation by activation of the cellular enzymes of the RNaseH family and consequently block gene expression [22]. The ONs with therapeutic potential include aptamers, transcription factor-binding decoy ONs, ribozymes, triplex-forming ONs, immunostimulatory CpG motifs, antisense ONs, and antagomirs.

CPPs have been used for the delivery of ONs by using either a covalent linkage or a non-covalent linkage with the therapeutic agent. They have been used to mediate the delivery of PNAs and PMOs through covalent linkage [33]. The formation of efficient non-covalent complexes comprising CPPs and both charged and uncharged steric block oligonucleotides like 2'-*O*-methyl, LNA, PNA and charged PNA derivatives, has also been described [34].

PNA–CPP conjugates were first demonstrated to block the expression of the galanin receptor mRNA in human Bowes cells by a 21-mer PNA coupled to Penetratin or Transportan by Langel and group in 1998 [22]. In a different study, a model amphipathic peptide (MAP) conjugated to a PNA complementary to the nociceptin/orphanin FQ receptor mRNA was shown to improve cellular uptake and steric block effect in both CHO cells and neonatal rat cardiomyocytes [35].

The efficacy of PNA and PMO coupled to CPPs was only significant in the presence of endosomolytic agents such as chloroquine and calcium ions [36,37,38]. Since most of the existing endosomolytic agents are too toxic to be considered for *in vivo* applications, CPP-based strategies that are efficient in the absence of PNA and PMO such as co-treatment with endosome-disrupting peptides are currently being explored [39,40].

### 3.4. Protein Delivery

Certain proteins are currently being used as therapeutic agents for the treatment of various diseases. CPPs are usually coupled to proteins through covalent bonds or through fusion constructs except Pep-1, a CPP that forms non-covalent complexes [16]. There is evidence that CPPs are able to facilitate the delivery of proteins into a wide variety of cells both *in vitro* and *in vivo*; Dowdy and group revealed that the delivery of these therapeutic proteins into tissues and across the blood-brain barrier was severely limited by the size and biochemical properties of the proteins. An intraperitoneal injection of the 120-kilodalton beta-galactosidase protein, fused to the protein transduction domain from the human immunodeficiency virus TAT protein, resulted in delivery of the biologically active fusion protein to all tissues in mice, including the brain [41]. CPPs therefore constitute a powerful tool that could be used to facilitate the delivery of protein-based therapeutics in pathological conditions, such as cancer, inflammatory diseases, oxidative stress-related disorders, diabetes and brain injury [36,42]. However, protein stability relies on weak non-covalent interactions between secondary, tertiary and quaternary structures, which must be preserved throughout the delivery process [43]. This makes proteins vulnerable therapeutic agents with short *in vivo* half-lives and poor bioavailability. To improve the efficiency of delivery of proteins into cells, different types of lipid- and polymer-based vectors including liposomes, microparticles and nanoparticles have been used for protein delivery, but with relatively poor efficiency [44].

### 3.5. Delivery of Drug Carriers

Liposomes, nanoparticles and other different types of pharmaceutical nanocarriers have been used to increase the stability of drugs, modulate their pharmacokinetics and biodistribution, improve their efficacy while decreasing side-effects [21]. However, the intracellular delivery of these large molecules remains a challenge because of their three-dimensional structure, spatial occupation and hydrophilic/hydrophobic nature.

CPPs have been used to functionalize these vectors with a view to increasing the cellular uptake of the encapsulated therapeutic agents [45]. CPPs have been shown to offer the opportunity to deliver therapeutic molecules that are even 200 times larger than the current bioavailability size restriction. In one such study Torchilin prepared targeted long-circulating PEGylated liposomes and PEG-phosphatidylethanolamine (PEG-PE)-based micelles possessing several functionalities [21]. These liposomes and micelles were modified with TATp moieties attached to the surface of the nanocarriers by using TATp-short PEG-PE derivatives. This made them degradable by inserting the pH-sensitive hydrazine bond between PEG and PE (PEG-Hz-PE) whose cleavability by acidic hydrolysis makes them acquire the ability to be effectively internalized and released. These can be considered as an important step in the development of tumor-specific stimuli-sensitive delivery systems.

Dextran-coated superparamagnetic iron oxide particles (CLIO) coupled with TATp, were demonstrated to provide efficient labeling of cells, and could serve as a tool for magnetic resonance imaging (MRI). The uptake of the TATp-CLIO nanoparticles by cells was shown to be about 100-fold higher than that of the non-modified iron oxide particle [21]. Other studies reveal that modifying the surface of nanoparticles with CPPs, enhances the cell permeability of nanoparticulate-based therapeutics [46]. This is further proved by the CPP-mediated delivery of bioactive compounds into model organisms for cancer, cardiomyopathy, stroke, muscular dystrophy and viral infections [47,48]. For example PsorBanR, a cyclosporin–poly-arginine conjugate formulated as a topical treatment for psoriasis, and KAI-9803, a PKC (protein kinase C) δ peptide inhibitor–TAT conjugate for the treatment of acute MI (myocardial infarction) [49].

A family of cell-penetrating peptides named Vectocell^®^ peptides [also termed DPVs (Diatos peptide vectors)] originating from human heparin binding proteins and/or anti-DNA antibodies with both enhanced and safe cell penetration characteristics have been identified. These new peptidic sequences are reported to deliver small and large active molecules inside cells that would otherwise have limited or no bioavailability [45].

### 3.6. CPP as Active Pharmaceutical Ingredients

CPPs have been reported to act as active pharmaceutical ingredients when used alone. A TAT peptide containing a cysteine residue at its C-terminal (TAT-C) was shown to be able to inhibit infection by irreversibly inactivating virions exposed to the tat-C prior to cell infection, blocking entry of cell-adsorbed viruses, or inducing a state of resistance to infection in cells pretreated with TAT-C [50]. The mechanism of the antiviral activity is yet to be elaborated. This has set the stage for exploring the therapeutic activity of CPPs as active pharmaceutical ingredients

## 4. Advances in Cell Penetrating Peptide Development

TAT and penetratin, the first CPPs to be described, paved the way to the discovery of other naturally occurring CPPs such as the herpes virus tegument protein VP22 and the cell wall protein-derived peptide inv3 from *Mycobacterium tuberculosis* [51], Chimaeric CPPs such as Transportan (a chimera of the neuropeptide galanin and the wasp venom toxin mastoparan) and totally synthetic CPPs such as the model amphipathic peptide (MAP) or arginine oligomers [52]. All these have been designed and are routinely used.

Advancements have resulted in the combination of a tumor homing peptide with a cell-penetrating peptide. This could yield a chimeric peptide with tumor cell specificity that could carry therapeutic molecules into the cells. In a previous study involving the use of linear breast tumor homing peptide, CREKA, in conjunction with a cell-penetrating peptide, pVEC, it was demonstrated that CREKA–pVEC is a suitable vehicle for targeted intracellular delivery of a DNA alkylating agent, chlorambucil. The chlorambucil–peptide conjugate was shown *in vitro* to kill cancer cells faster than the anticancer drug alone [53].

Several reviews have elaborated on examples of novel drug delivery systems involving CPPs. All these systems are geared towards targeting a specific cell or tissue. Many of the strategies described have proved successful in *in vivo* experiments. This has led to preclinical and clinical studies of CPP-based delivery strategies [54]. Such studies include:

a.) PsorBan^®^ a cyclosporine-poly-arginine conjugate for the topical treatment of psoriasis was the first CPP mediated therapeutic agent which entered phase II trials in 2003 (CellGate, Inc.)., Delcasertib as KAI-9803 was recently tested by Kai Pharmaceutical as a TAT-protein kinase C inhibitor peptide modulator of protein kinase C for acute myocardial infarction and cerebral ischemia, and orally administrated cyclosporine A (CsA), effective against a broad range of inflammatory skin diseases including psoriasis, are examples of ongoing preclinical studies on effective delivery strategies [55]. Conjugation of a CPP, heptaarginine with CsA through a linker designed to release the active compound at the pH of the tissue has been shown to enhance its topical absorption, inhibiting cutaneous inflammation [56].
b.) Avi Biopharma is working on the clinical development of CPPs for the *in vivo* steric block splicing correction using 6-aminohexanoic acid spaced oligoarginine [(RAhx-R)_4_]. It consists of a Morpholino oligo conjugated with the CPP [(RXR)_4_-XB-CPP]. The goal of this conjugate is to prevent eventual blockage of a transplanted vein after cardiovascular bypass surgery [57]. Several other companies including Traversa Inc., and Panomics Inc. are also evaluating CPPs in preclinical and clinical trials in addition to other molecules conjugated to CPPs which are being optimized [58].

Phosphorodiamidate Morpholino Oligomers (PMOs) are antisense DNA oligonucleotide analogues with a backbone composed of morpholine rings joined by uncharged phosphodiamidate linkages in place of the sugar and anionic phosphodiester linkage of DNA. They are water-soluble, nuclease-resistant, and have an uncharged backbone, which can interact weakly with serum and cellular proteins thereby reducing toxicity. They therefore offer great promise in clinical applications [59]. As PMOs do not efficiently enter cells on their own, investigators have been routinely linking them to CPPs to promote their uptake into virus-infected cells and enhance their antisense efficacy.

PMO-technology to inhibit viral infections has been exploited as extensively reviewed by Stein [60] and recently discussed by Moulton and Jiang [61] where a very potent CPP-PMO conjugate strongly inhibited HSV-1 replication in cell cultures by reducing viral protein expression. It was reported to have been able to suppress the replication of several HSV-1 strains, including an acyclovir-resistant strain.

In addition to improving PMO cellular uptake, the CPP moiety has also been reported to intensify PMO antisense activity against Ebola virus (EBOV) 10- to 100-fold in cell-free translation assays. These results are consistent with other findings. The authors proposed that the arginine-rich peptides enhance RNA-PMO binding affinity, thereby increasing specific antisense activity [62].

Due to poor cellular uptake of the uncharged peptide-nucleic acids (PNAs), Pandey and co-workers covalently conjugated PNA at its *N* terminal to various cell-penetrating peptides, including Transportan, TAT and penetrain, via disulfide bridges. Upon coupling to carrier peptides, the PNA therapeutic agents were rapidly taken up by different types of human cells in culture. When added to culture medium, these anti-HIV-1 PNA–CPP conjugates effectively inhibited HIV-1 replication, tat-dependent trans-activation and viral production by infected cells. No significant decrease was observed with the unconjugated PNA, emphasizing the great promise held by these conjugates as antiviral agents [63].

Preliminary toxicity, immunological and pharmacokinetic studies in mice for the anti-HIV-1 PNATAR-Penetratin conjugate have just started. Investigating the tissue distribution and clearance of 125I-labeled PNATAR, PNATAR-penetrain and PNATAR-TAT; Ganguly’s group reported the distribution of the conjugates throughout the mouse major internal organs when administered by oral route as well as their slow release and clearance from different organs [64]. The unconjugated PNATAR was noted to display a similar tissue distribution and clearance profile although the extent of its uptake was lower than its CPP conjugate. This calls for further investigation.

The use of CPPs to deliver siRNAs into cells has received rather less attention especially for antiviral siRNAs due to the fact that siRNAs is less amenable to CPP delivery as a result of charge interactions between the peptide and the siRNA which results in inefficient endosomal escape of the conjugates. However a cell-permeable CPP-siRNA conjugate targeting hepatitis C virus 5’ untranslated region has been formulated [63]. The cellular uptake and antiviral effect of this CPP-siRNA conjugate were reported to be as effective as transfection with lipofectamine in Huh-7 cell culture.

In addition to targeting HIV-1, CPP-mediated protein delivery has also been used to inhibit human papillomavirus type 18 (HPV-18) in cell culture. Delivery of artificial zinc-finger proteins (AZPs) into cultured cells by expressing AZPs in fusion with the 9R-peptide has been reported. These AZP-9R conjugates strongly reduced HPV-18 replication to 3% at 250 nM while non-conjugated AZP showed only 12% reduction. PTD4 conjugates were also tested but proved to be less effective than AZPs fused to 9R [65].

PNA-CPP complexes have also been investigated for virucidal activity microbicides [63]. The study reported that pre-incubation of HIV-1 virions with these molecules rendered them noninfectious and blocked further cell infection. It is suggested that the PNA-CPP may have altered or disrupted the viral envelope through the interaction of the CPP moiety with the viral lipid bilayer, thereby inhibiting host cell infection [52].

However CPPs have their limitations as drug delivery carriers. The *in vitro* uptake studies revealed high cellular uptake values, but no specificity toward any of the cell lines. Their biodistribution in PC-3 tumor-bearing nude mice showed a high transient accumulation in well-perfused organs and a rapid clearance from the blood [55]. Data obtained in the study reveal that CPPs readily penetrate into most organs and show rapid clearance from the circulation. In view of this, it is suggested that CPPs are suitable as drug carriers for *in vivo* application provided that their cell specificity and pharmacokinetic properties are also considered in design and development of CPP-based drug delivery systems.

## 5. Chemical Modification of CPP for Enhanced Delivery

### 5.1. Amino Acid Substitution

Apart from functional group modification of amino acids in the peptide chain, modification of cell penetrating peptides have also been shown to involve substitution of amino acids to achieve variability of the peptide properties such as hydrophobicity or cationic nature. This strategy has been noted to result in increased intracellular internalization by certain CPPs. Kaeko *et al.* conducting a comprehensive search for novel CPPs using an *in vitro* virus library of peptides consisting of 15 amino acids [19] and reported improved intracellular translocation efficiency at low concentrations due to the influence of cationic amino acids. Since the amino acid Arginine has a stronger affinity to the cell surface, a desirable modification in this case involved substituting another amino acid such as Lysine in the peptide chain with Arginine. Such substitutions were reported [66] to have remarkably improved intracellular translocation even at low concentration. Substitution (of amino acids) with Histidine residue is also a promising option as this could provide endosomal disruption by the “proton sponge effect” of Histidine residues in the acidic endosomal compartment as previously reported for LAH4; an amphiphatic peptide rich in Alanine and Leucine. It bears Lysines at its ends to condense DNA and Histidines inside the sequence which favours endosomal escape. LAH4/DNA polyplexes were reported to be able to transfect cells in the absence of serum. From a series of LAH4 derivatives, high transfection efficiencies were obtained only with peptides containing four to five Histidine residues in the central region of the peptide sequence [67].

Increasing amino acids like Tryptophan which has the tendency to be buried in the cell membrane [68] improves CPP hydrophobic interaction. It has been confirmed by Kaeko *et al.* that in low peptide concentrations, two Trp residues were not enough to initiate effective translocation, but three Trp enhanced cellular uptake. However, when the number of Tryptophan residues increased to four the intracellular translocation activity decreased [19]. This may be due to a decrease in solubility of the peptide as a result of the increased number of Tryptophan residues. Therefore, the increase of the number of Trp residues may be ideal but to a certain limit and such modifications should be carried out with strict consideration of the impact of amino acid substitution to the physicochemical parameters, especially changes in dissolution properties.

### 5.2. Functional Group Modification

Functional group modification of the amino acids in the peptide usually involves the formation of masking groups or linkages to highly reactive sites. In either case, the peptide bonds formed should be labile and easily broken for regeneration of the initial cell penetrating peptide by simple variation of physiological conditions.

Helicity of the peptide chain can be exploited for modification. For example through hydrocarbon stapling the overall cell penetrating peptide’s alpha helicity could further be stabilized. This is derived from the fact that the α-helix, as a major structural motif of proteins, frequently mediates intracellular protein-protein interactions that govern many biological pathways [69].

Fei and co workers reported a modification strategy to decrease the non-specific binding and uptake of a CPP, model amphipathic peptide (MAP, for use as a potential targeted drug carrier [68]. This was illustrated using (S)-a-(2’-pentenyl)alanine containing olefin-bearing tethers to generate an all hydrocarbon “staple” by ruthenium-catalyzed olefin metathesis. The (S)-a-(2’-pentenyl)alanine peptides were made to flank three (substitution positions l and l + 4) or six (l and l + 7) amino acids within the peptide, so that reactive olefinic residues would reside on the same face of the a-helix. The modified hydrocarbon-stapled peptides were helical, relatively protease-resistant, and cell-permeable peptides that bound with increased affinity for the target [70]. Such hydrocarbon stapling could provide a useful strategy for therapeutic modulation of protein-protein interactions.

In another study the modification of MAP with citraconic anhydride (CA) blocks, the epsilon-amino groups of the Lysine residues formed acid-labile amide linkages. This was confirmed by analysis for internalization in HeLa cells which revealed that 80.8 ± 2.2% of the amino groups were modified in the CA-MAP conjugate. In the study a MAP conjugate was designed to contain acid-labile modifications to mask the cationic charge, and therefore decreasing the non-specific binding and uptake [71,72]. After binding and internalization, the acidic pH in the endosomes was realized to facilitate the cleavage of the acid-labile attachments and hence release of MAP. The combination of CA-MAP with a targeting molecule (e.g., folic acid) offers significant improvement in the use of CPPs in targeted drug delivery.

## 6. Conclusions

Over the years of research focus has been on how to optimize drug delivery systems to increase both cell specificity and drug delivery efficiency. The use of cell penetrating peptides demonstrates the possibility that these drug systems can be improved. Efficient targeting by utilizing multi-functional polymer vehicles with capabilities for endosome disruption and nuclear penetration if appropriately considered can yield cell-penetrating peptides with high cellular specificity. CPP modification presents viable options that such delivery systems can further be improved. This could result in biomolecules that could be triggered spatially and temporally within a cell transport pathway hence truly improving drug therapy. There is therefore a need to develop assays to compare the different existing methods in order to study and optimize modified CPP-mediated delivery.

## References

[1] De Coupade C., Fittipaldi A., Chagnas V., Michel M., Carlier S., Tasciotti E., Darmon A., Ravel D., Kearsey J., Giacca M., Cailler F. (2005). Novel human-derived cell-penetrating peptides for specific subcellular delivery of therapeutic biomolecules. Biochem. J..

[2] Veerle K., Cornelissen B. (2010). Targeting the tumour: Cell penetrating peptides for molecular imaging and radiotherapy. Pharmaceuticals.

[3] Nathan S., Abhijit M., Ghee H.L., Gerard W.C.L. (2010). Arginine-rich cell-penetrating peptides. FEBS Lett..

[4] Heitz F., Morris M.C., Divita G. (2009). Twenty years of cell-penetrating peptides: From molecular mechanisms to therapeutics. Br. J. Pharmacol..

[5] Deshayes S., Morris M.C., Divita G., Heitz F. (2005). Cell-penetrating peptides: Tools for intracellular delivery of therapeutics. Cell. Mol. Life Sci..

[6] Snyder E.L., Dowdy S.F. (2005). Recent advances in the use of protein transduction domains for the delivery of peptides, proteins and nucleic acids *in vivo*. Expert Opin. Drug Deliv..

[7] Fisher L., Soomets U., Cortés Toro V., Chilton L., Jiang Y., Langel U., Iverfeldt K. (2004). Cellular delivery of a double-stranded oligonucleotide NFkappaB decoy by hybridization to complementary PNA linked to a cell-penetrating peptide. Gene Ther..

[8] Zatsepin T.S., Turner J.J., Oretskaya T.S., Gait M.J. (2005). Conjugates of oligonucleotides and analogues with cell penetrating peptides as gene silencing. Curr. Pharm. Des..

[9] Pujals S., Fernandez-Carneado J., Lopez-Iglesias C., Kogan M.J., Giralt E. (2006). Mechanistic aspects of CPP-mediated intracellular drug delivery: Relevance of CPP self-assembly. Biochim. Biophys. Acta.

[10] El-Andaloussi S., Holm T. Langel (2005). Cell-penetrating peptides: Mechanism and applications. Curr. Pharm. Des..

[11] Murriel C.L., Dowdy S.F. (2006). Influence of protein transduction domains on intracellular delivery of macromolecules. Expert Opin. Drug Deliv..

[12] Schwarze S.R., Hruska K.A., Dowdy S.F. (2000). Protein transduction: Unrestricted delivery into all cells?. Trends Cell Biol..

[13] Abes S., Turner J., Ivanova G.D., Owen D., Williams D., Arzumanov A. (2007). Efficient splicing correction by PNA conjugation to an R6-Penetratin delivery peptide. Nucleic Acids Res..

[14] Meade B.R., Dowdy S.F. (2007). Exogenous siRNA delivery using peptide transduction domains/cell penetrating peptides. Adv. Drug Deliv. Rev..

[15] Morris M.C., Deshayes S., Heitz F., Divita G. (2008). Cell-penetrating peptides: From molecular mechanisms to therapeutics. Biol. Cell.

[16] Gros E., Deshayes S., Morris M.C., Aldrian-Herrada G., Depollier J., Heitz F. (2006). A non-covalent peptide-based strategy for protein Peptide-based drug delivery technology and peptide nucleic acid delivery. Biochim. Biophys. Acta.

[17] Munoz-Morris M.A., Heitz F., Divita G., Morris M.C. (2007). The peptide carrier Pep-1 forms biologically efficient nanoparticle complexes. Biochem. Biophys. Res. Commun..

[18] Langel U. (2006). Handbook of Cell-Penetrating Peptides.

[19] Kaeko K., Hiroshi N., Shusei U., Akiyoshi F. (2010). Isolation of novel cell-penetrating peptides from a random peptide library using *in vitro* virus and their modifications. Int. J. Mol. Med..

[20] Torchilin V.P. (2008). Cell penetrating peptide—Modified pharmaceutical nanocarriers for intracellular drug and gene delivery. Biopolymers (Pept. Sci.).

[21] Trabulo S., Cardoso A.L., Mano M., de Lima M.C.P. (2010). Cell-penetrating peptides—Mechanisms of cellular uptake and generation of delivery systems. Pharmaceuticals.

[22] Glover D.J., Lipps H.J., Jans D.A. (2005). Towards safe, non-viral therapeutic gene expression in humans. Nat. Rev. Genet..

[23] Kilk K., El-Andaloussi S., Järver P., Meikas A., Valkna A., Bartfai T. (2005). Evaluation of Transportan 10 in PEI mediated plasmid delivery assay. J. Control. Release.

[24] Martin M.E., Rice K.G. (2007). Peptide-guided gene delivery. AAPS J..

[25] Endoh T., Ohtsuki T. (2009). Cellular siRNA delivery using cell-penetrating peptides modified for endosomal escape. Adv. Drug Deliv. Rev..

[26] Kim W.J., Christensen L.V., Jo S., Yockman J.W., Jeong J.H., Kim Y.H. (2006). Cholesteryl oligoarginine delivering vascular endothelial growth factor siRNA effectively inhibits tumor growth in colon adenocarcinoma. Mol. Ther..

[27] Crombez L., Charnet A., Morris M.C., Aldrian-Herrada G., Heitz F., Divita G. (2007). A non-covalent peptide-based strategy for siRNA delivery. Biochem. Soc. Trans..

[28] Nguyen Q.N., Chavli R.V., Marques J.T., Conrad P.G., Wang D., He W. (2006). Light controllable siRNAs regulate gene suppression and phenotypes in cells. Biochim. Biophys. Acta.

[29] Zeineddine D., Papadimou E., Chebli K., Gineste M., Liu J., Grey C. (2006). Oct-3/4 dose dependently regulates specification of embryonic stem cells toward a cardiac lineage and early heart development. Dev. Cell.

[30] Kumar P., Wu H., McBride J.L., Jung K.E., Kim M.H., Davidson B.L. (2007). Transvascular delivery of small interfering RNA to the central nervous system. Nature.

[31] Ezzat K., Andaloussi S.E.L., Zaghloul E.M., Lehto T., Lindberg S., Moreno P.M.D., Viola J.R., Magdy T., Abdo R., Guterstam P., Sillard R., Hammond S.M., Wood M.J.A., Arzumanov A.A., Gait M.J., Smith C.I.E., Hällbrink M., Langel Ü. (2011). PepFect 14, a novel cell-penetrating peptide for oligonucleotide delivery in solution and as solid formulation. Nucleic Acids Res..

[32] Lebleu B., Moulton H.M., Abes R., Ivanova G.D., Abes S., Stein D.A., Iversen P.L., Arzumanov A.A., Gait M.J. (2008). Cell penetrating peptide conjugates of steric block oligonucleotides. Adv. Drug Deliv. Rev..

[33] Laufer S.D., Recke A.L., Veldhoen S., Trampe A., Restle T. (2009). Noncovalent peptide-mediated delivery of chemically modified steric block oligonucleotides promotes splice correction: Quantitative analysis of uptake and biological effect. Oligonucleotides.

[34] Oehlke J., Wallukat G., Wolf Y., Ehrlich A., Wiesner B., Berger H., Bienert M. (2004). Enhancement of intracellular concentration and biological activity of PNA after conjugation with a cell-penetrating synthetic model peptide. Eur. J. Biochem..

[35] Abes S., Williams D., Prevot P., Thierry A., Gait M.J., Lebleu B. (2006). Endosome trapping limits the efficiency of splicing correction by PNA-oligolysine conjugates. J. Control. Release.

[36] Shiraishi T., Pankratova S., Nielsen P.E. (2005). Calcium ions effectively enhance the effect of antisense peptide nucleic acids conjugated to cationic TAT and oligoarginine peptides. Chem. Biol..

[37] Wolf Y., Pritz S., Abes S., Bienert M., Lebleu B., Oehlke J. (2006). Structural requirements for cellular uptake and antisense activity of peptide nucleic acids conjugated with various peptides. Biochemistry.

[38] Eguchi A., Dowdy S.F. (2009). siRNA delivery using peptide transduction domains. Trends Pharmacol. Sci..

[39] Endoh T., Ohtsuki T. (2009). Cellular siRNA delivery using cell-penetrating peptides modified for endosomal escape. Adv. Drug Deliv. Rev..

[40] Deshayes S., Morris M., Heitz F., Divita G. (2008). Delivery of proteins and nucleic acids using a non-covalent peptide-based strategy. Adv. Drug Deliv. Rev..

[41] Schwarze S.R., Ho A., Vocero-Akbani A., Dowdy S.F. (1999). *In vivo* protein transduction: Delivery of a biologically active protein into the mouse. Science.

[42] Tan M.L., Choong P.F., Dass C.R. (2009). Recent developments in liposomes, microparticles and nanoparticles for protein and peptide drug delivery. Peptides.

[43] Clayton R., Ohagen A., Nicol F., Del Vecchio A.M., Jonckers T.H.M., Goethals O., van Loock M., Michiels L., Grigsby J., Xu Z., Zhang Y.P., Gutshall L.L., Cunningham M., Jiang H., Bola S., Sarisky R.T., Hertogs K. (2009). Sustained and specific *in vitro* inhibition of HIV-1 replication by a protease inhibitor encapsulated in gp120 - targeted liposomes. Antivir. Res..

[44] McCarthy J.R., Weissleder R. (2008). Multifunctional magnetic nanoparticles for targeted imaging and therapy. Adv. Drug Deliv. Rev..

[45] Koch A.M., Reynolds F., Merkle H.P., Weissleder R., Josephson L. (2005). Transport of surface-modified nanoparticles through cell monolayers. Chembiochem.

[46] Lee H., Jefferies R., Watt P., Hopkins R., Sotzik F., Reid S., Armson A., Boxell A., Ryan U. (2008). *In vitro* analysis of the TAT protein transduction domain as a drug delivery vehicle in protozoan parasites. Exp. Parasitol..

[47] Bian J., Popovic Z.B., Benejam C., Kiedrowski M., Rodriguez L.L., Penn M.S. (2007). Effect of cellbased intercellular delivery of transcription factor GATA4 on ischemic cardiomyopathy. Circ. Res..

[48] Marcella F., Farzin F., Nagy H., Farooq M., Joop G., Mahvash T. (2009). Delivery of therapeutic proteins as secretable TAT fusion products. Mol. Ther..

[49] Mino T., Mori T., Aoyama Y., Sera T. (2008). Cell-permeable artificial zinc-finger proteins as potent antiviral drugs for human papillomaviruses. Arch. Virol..

[50] de Coupade C., Fittipaldi A., Chagnas V., Michel M., Carlier S., Tasciotti E., Darmon A., Ravel D., Kearsey J., Giacca M., Cailler F. (2005). Novel human-derived cell-penetrating peptides for specific subcellular delivery of therapeutic biomolecules. Biochem. J..

[51] Harrison S.D., Chen L. (2007). Cell-penetrating peptides in drug development: Enabling intracellular targets. Biochemical Society Transactions.

[52] Jearawiriyapaisarn N., Moulton H.M., Buckley B., Roberts J., Sazani P., Fucharoen S., Iversen P.L., Kole R. (2008). Sustained dystrophin expression induced by peptide-conjugated morpholino oligomers in the muscles of mdx mice. Mol. Ther..

[53] Sarko D., Beijer B., Garcia Boy R., Nothelfer E.M., Leotta K., Eisenhut M., Altmann A., Haberkorn U., Mier W. (2010). The pharmacokinetics of cell-penetrating peptides. Mol. Pharm..

[54] Martín I., Teixidó M., Giralt E. (2010). Building cell selectivity into CPP-mediated strategies. Pharmaceuticals.

[55] Chen L., Harrison S.D. (2007). Cell-penetrating peptides in drug development: Enabling intracellular targets. Biochem. Soc. Trans..

[56] Lebleu B., Moulton H.M., Abes R., Ivanova G.D., Abes S., Stein D.A., Iversen P.L., Arzumanov A.A., Gait M.J. (2008). Cell penetrating peptide conjugates of steric block oligonucleotides. Adv. Drug Deliv. Rev..

[57] Sebbage V. (2009). Cell-penetrating peptides and their therapeutic applications. Biosci. Horiz..

[58] Juliano R.L., Alam R., Dixit V., Kang H.M. (2009). Cell-targeting and cell-penetrating peptides for delivery of therapeutic and imaging agents. Wiley Interdiscip. Rev. Nanomed. Nanobiotechnol..

[59] Summerton J.E. (2007). Morpholino, siRNA, and S-DNA compared: Impact of structure and mechanism of action on off-target effects and sequence specificity. Curr. Top. Med. Chem..

[60] Stein D.A. (2008). Inhibition of RNA virus infections with peptide-conjugated morpholino oligomers. Curr. Pharm. Des..

[61] Moulton J.D., Jiang S. (2009). Gene knockdowns in adult animals: PPMOs and vivo-morpholinos. Molecules.

[62] Swenson D.L., Warfield K.L., Warren T.K., Lovejoy C., Hassinger J.N., Ruthel G., Blouch R.E., Moulton H.M., Weller D.D., Iversen P.L., Bavari S. (2009). Chemical modifications of antisense morpholino oligomers enhance their efficacy against Ebola virus infection. Antimicrob. Agents Chemother..

[63] Tripathi S., Chaubey B., Barton B.E., Pandey V.N. (2007). Anti HIV-1 virucidal activity of polyamide nucleic acid-membrane transducing peptide conjugates targeted to primer binding site of HIV-1 genome. Virology.

[64] Ganguly S., Chaubey B., Tripathi S., Upadhyay A., Neti P., Howell R.W., Pandey V.N. (2008). Pharmacokinetic analysis of polyamide nucleic-acid-cell penetrating peptide conjugates targeted against HIV-1 transactivation response element. Oligonucleotides.

[65] Amand H.L., Fant K., Nordén B., Esbjörner E.K. (2008). Stimulated endocytosis in Penetratin uptake: Effect of arginine and lysine. Biochem. Biophys. Res. Commun..

[66] Mason A.J., Leborgne C., Moulay G., Martinez A., Danos O., Bechinger B. (2007). Optimising histidine rich peptides for efficient DNA delivery in the presence of serum. J. Contr. Rel..

[67] Henriques S.T., Castanho M.A. (2008). Translocation or membrane disintegration? Implication of peptide-membrane interactions in pep-1 activity. J. Pept. Sci..

[68] Fei L., Zaro J., Shen W.-C. (2009). Acid-labile Modification of a Cell Penetrating Peptide for Use in Targeted Drug Delivery. Contributed Papers: Drug Delivery Biopharmaceutics/Other,/AAPS2009-002238. http://www.aapsj.org/abstracts/AM.

[69] Veerle K., Cornelissen B. (2010). Targeting the tumour: Cell penetrating peptides for molecular imaging and radiotherapy. Pharmaceuticals.

[70] Delcroix M., Riley L.W. (2010). Cell-penetrating peptides for antiviral drug development. Pharmaceuticals.

[71] Gupta B., Levchenko T.S., Torchilin V.P. (2005). Intracellular delivery of large molecules and small particles by cell-penetrating proteins and peptides. Adv. Drug Deliv. Rev..

[72] Mäe M., Myrberg H., El-Andaloussi S., Langel Ü. (2009). Design of a tumor homing cell-penetrating peptide for drug delivery. Int. J. Pept. Res. Ther..

